# Use of maternal health services among women in the ethnic rural areas of western China

**DOI:** 10.1186/s12913-019-3996-2

**Published:** 2019-03-19

**Authors:** Yuju Wu, Huan Zhou, Qingzhi Wang, Min Cao, Alexis Medina, Scott Rozelle

**Affiliations:** 1 Department of Health and Social Behavior Science, West China School of Public Health, Sichuan University, No.16, section 3, South Renmin Road, Chengdu City, Sichuan Province 610041 People’s Republic of China; 2 The Freeman Spogli Institute for International Studies, Stanford University, Stanford, CA USA

**Keywords:** Maternal health services, Antenatal care, Ethnic areas, Western China

## Abstract

**Background:**

The use of maternal health services can markedly promote the maternal health and safety, but there has been a low utilization rate in the ethnic rural areas of western China. Furthermore, the correlated factors have not been well studied. This study aims to assess factors related to the use of maternal health services among women in these areas.

**Methods:**

A cross-sectional study of 68 villages in China’s western Sichuan province was conducted in September 2014. All qualifying women from each sample village were involved. A structured questionnaire was administrated in households through face-to-face interviews by trained enumerators to obtain information of use of maternal health services and related factors. Structural equation modeling (SEM) was used to evaluate the direct and indirect relationships between use of maternal health services and correlated factors.

**Results:**

A total of 760 women from 68 villages were enrolled. The proportion of antenatal care (ANC), hospital delivery and postpartum visits were 68.94, 48.29 and 28.42% respectively. The SEM analysis demonstrated that social economic status (SES) (*β*= − 0.75, *β*< 0.01), ANC (*β*=0.13, *β*< 0.01), and time from home to the nearest hospital (*β*= − 0.09, *β*< 0.05), were positively correlated to hospital delivery and postpartum care visits, while maternal care knowledge and perceived quality of hospital care did not have direct correlation. For ANC, SES (*β*= − 0.36, *β*< 0.01), time from home to the nearest hospital (*β*= − 0.13, *β*< 0.05), knowledge on maternal care (*β*=0.12, *β*< 0.01) and perceived quality of hospital care (*β*=0.10, *β*< 0.01) were all directly correlated factors. Treating ANC as an intermediate variable showed the indirect relationship that perceived quality of hospital care (*β*=0.01, *β*< 0.01) and maternal care knowledge (*β*=0.02, *β*< 0.01) had with hospital delivery and postpartum care rates.

**Conclusions:**

Use of maternal health services is low among women in ethnic rural areas. ANC has important direct and intermediate effects on subsequent use of hospital delivery and postpartum care. Improving ANC behavior should be a priority of maternal health care reforms. Given the long travel times for these women, reforms must also prioritize breaking down practical barriers that prevent this population from accessing care.

**Electronic supplementary material:**

The online version of this article (10.1186/s12913-019-3996-2) contains supplementary material, which is available to authorized users.

## Background

The use of maternal health services is integral to reducing maternal mortality and ensuring mother and child safety [1]. The Report on Women and Children’s Health Development in China (2011) recommends that women seek maternal health services multiple times, including at least one early pregnancy test, two appointments during mid-pregnancy, two appointments during late pregnancy, hospital delivery, and postpartum visits [2]. Numerous studies have shown that an effective prenatal examination, hospital delivery, and postpartum visits can reduce the incidence of maternal diseases, low birth weight, maternal and infant mortality, and postpartum depression [3–5]. However, the use of maternal health service was delayed or even absent in some areas of developing countries. The research from Kenya indicated that 14% of young woman did not attend ANC and 76% were later than recommendation [6]. More than half of pregnant women gave birth at home in rural areas of India [7]. Increasing the use of maternal health care services is therefore essential for improving the health of women and children in these areas.

Since the founding of the People’s Republic of China, numerous measures have been implemented to improve the utilization rate of maternal health services and reduce mortality. For instance, one national project aimed at improving maternal health is focused on reducing maternal mortality and neonatal tetanus in Central and Western China [2]. In the 2009 Chinese Health System Reform (CHSR), the well-being of mothers and children is recognized as an important public health issue [8], and in recent years, maternal health services have come to be seen as one of the nation’s essential public health services [9]. National health statistics data show that the utilization rate of antenatal care (ANC) services, hospital deliveries, and postpartum visits were 52.7, 69.7, and 69.7% respectively in 1992, but by 2012 rates had increased to 95.0, 99.2, and 92.6% respectively. By 2012 the rate of maternal mortality had also dropped to 24.5/10,000 [10]. Urban-rural gaps in care have also begun to close; in 2013, the fifth national health service survey data showed that the hospital delivery rate was 96.8% in rural areas and 95.7% in urban areas [11].

While the gap in maternal health service utilization rates between urban and rural areas has gradually narrowed at the national level, rates in many rural areas remain alarmingly low. Only 63.5% of women of childbearing age in rural Shaanxi province attended an initial ANC visit within the first 12 weeks of their pregnancy [12]. In another study from 2012, 93.6% of pregnant women in rural areas of Ningxia province received antenatal care, but only 88.3% delivered their child in a hospital and 41.5% returned for postpartum visits [13].

Inadequacies in maternal health services are even more severe among minorities living in poor, rural regions. Sichuan province, located in the southwest mountainous region of China, has 55 ethnic minorities, of which the Yi is the largest [14]. In a 2011 study evaluating health services in rural minority areas of Sichuan province, the rate of ANC was 65.5%, and the rate of hospital delivery was only 33.6% [15]. A 2012 study showed that the use of maternal health services in the Yi community was extremely low, with a postpartum visit rate of only 22.9% [16]. Thus, public health policy reforms in China’s poor, rural, and minority areas must remain focused on increasing the use of maternal health services.

The main research question of the study asks what factors were significantly correlated with the utilization of maternal health services, and what were the associations between them. This study aims to investigate what factors influence maternal health service use in populations with low utilization rates in Sichuan, western China. To accomplish this goal, we surveyed 780 women living in poor, rural, predominantly Yi communities. Studies have indicated that age, ethnicity, educational level, beliefs, transportation accessibility, income, pregnancy knowledge, and health service structure are all factors associated with the use of maternal health services in rural areas [17–19]. However, the role of ANC for hospital delivery and postpartum visits was underexplored in the previous researches. In this paper we report survey results about the relationships between factors and outcomes by introducing a structural equation model (SEM) to analyze both direct and indirect relationships.

## Methods

### Study design

This cross-sectional study was conducted in September 2014 in five nationally designated impoverished counties in China’s western Sichuan province. These counties were selected because the United Nations Children’s Fund has focused their efforts on enhancing the township health centers and health outcomes of women in these areas. This study was part of a larger project on the use of maternal and infant health services in poor, rural, and minority areas of western China.

### Sampling methods and population

**Fig. 1:**
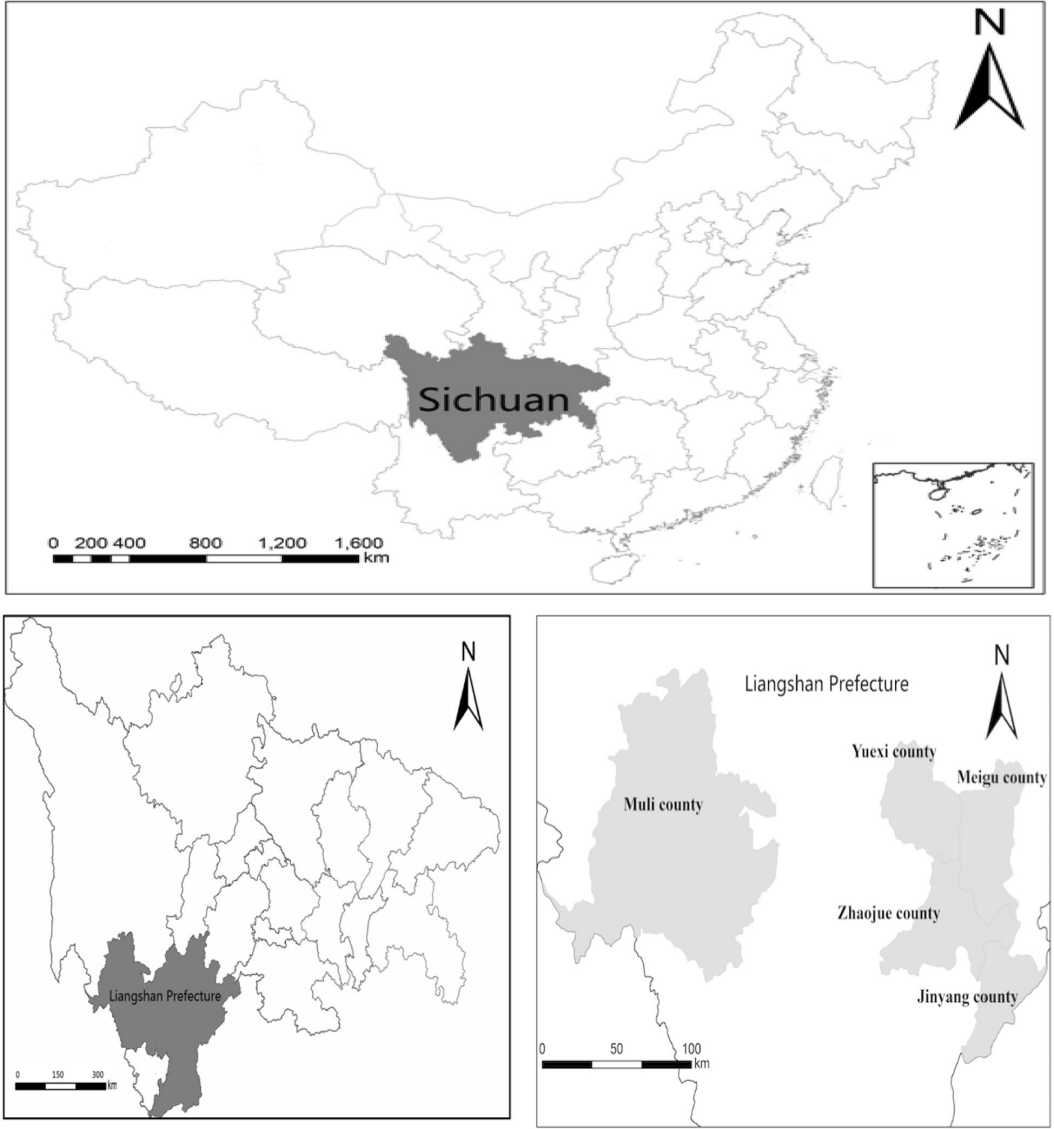
Map of the research areas: rural ethnic areas of the Sichuan province in western China. Copyright: author’s analysis of data from the Maternal Health Services survey, 2014

Our sample was selected from Liangshan Yi Autonomous Prefecture in Sichuan province, which is home to the largest Yi minority community in China. In the first stage, five nationally designated impoverished counties previously selected by the United Nations Children’s Fund were chosen. In the second stage, 68 towns with township health centers capable of offering maternal health services were identified. In the third stage, after excluding the villages where town governments were located, as well as those with populations of less than 800 people, 1 village from each sample town was randomly selected, forming a wide-scale survey of 68 sample villages. The selection pool included women who resided in the study area and had an infant between the ages of 3 and 24 months as of September 2014. Within each sample village, all the qualified households were involved in the survey. If participants had more than one pregnancy and delivery over the study period, information from all pregnancies was collected. The proportion of these woman, however, comprised less than 2% of the total sample. Moreover, most of them had different rates of utilization of maternal health services for different pregnancies. In total we obtained information from 780 households. Figure 1 shows the five counties of the study area.

### Data collection

A structured questionnaire was administrated in village households through face-to-face interviews by trained enumerators. Questionnaires asked for the following information:

1. Participant socioeconomic status (age, ethnicity, educational level, parity, family fixed assets, and occupation).
2. Perceived quality of hospital care (including the township health center and county hospital, hospital’s ability to provide maternal health services, perceived evaluation of technology, equipment, staff attitudes, and sanitation in the hospital).
3. Knowledge on maternal care (including questions on the risk of touching animals, harms of smoking, vaginal bleeding, bellyache, lower limb edema, irregular fetal movement, and the necessity and recommended number of ANC and postpartum visits). For each question the participant answered correctly, 1 point was added. The maximum possible score was 8.
4. Travel time to the nearest hospital capable of providing maternal health services. Note that this is only one measure of geographic proximity, and travel times cannot provide information about availability of transportation options.
5. Utilization of maternal health services (including ANC, hospital deliveries and postpartum visits). To ensure the accuracy of reported hospital deliveries, each village representative was asked to instruct participants to offer their child’s birth certificate (with the place of birth) before the interview.

Our questionnaire was developed for this study by experts in maternal and children health, epidemiology and health economics (Additional file 1). Before the actual data collection, the questionnaire was pre-tested on 20 women from villages that were in the study area (but, were not in villages that were eventually included in the study). This pretesting was done to assure the clarity of the concepts that we were studying. The reliability of scaling variables was assessed by calculating Cronbach’s coefficient alpha (α = 0.77), which indicates that our measures had fairly high levels of reliability.

### Setting up the model

**Fig. 2:**
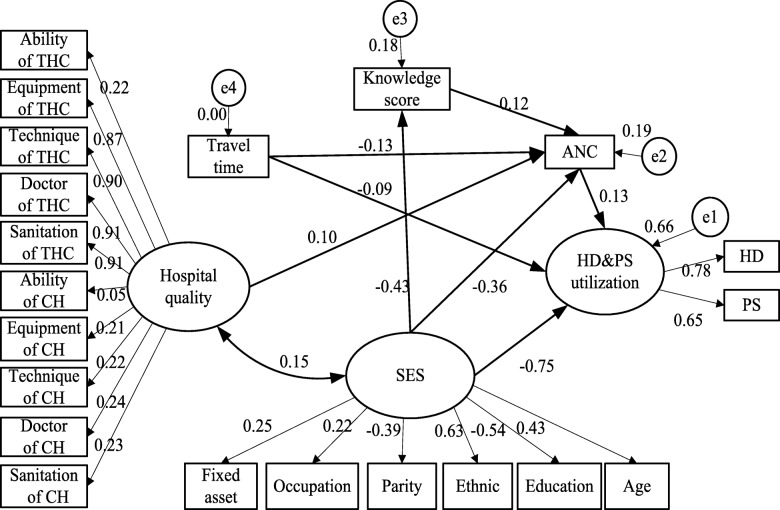
Structure of the basic model and modified model. Knowledge score = knowledge score on maternal care. HD&PS utilization = utilization of hospital delivery and postpartum visits. SES = women’s socioeconomic status; Hospital quality = perceived quality of hospital car. Travel time = the travel time from home to the nearest hospital

Structural equation modeling (SEM) integrates confirmatory factor analysis and linear regression methods, to test the rationality of a research hypothesis and reveal both direct and indirect relationships between variables through a path chart [20, 21]. In this study, SEM was conducted using two latent variables: SES and perceived quality of hospitals, and three measured variables: knowledge on maternal care, travel time to the nearest hospital, and ANC. Before setting up the model, we ran χ^2^ and t-tests to identify which of our input variables were significantly correlated with the use of maternal health services. Based on these results along with previous studies that revealed the use of maternal health services is impacted by participant attitudes and characteristics, quality of health services, and physical environment [22–25], we chose variables to set up the basic SEM model. Some studies also revealed that ANC use is positively correlated with hospital delivery, a relationship which was confirmed in our own results during univariate analysis [26, 27]. To further investigate the role of ANC as an intermediate variable affecting hospital delivery and postpartum care, we also included ANC as an input variable in our basic model (see Fig. 2). We looked for both direct and indirect correlations between all factors in the basic model.

### Statistical analysis

Statistical analysis was conducted using Stata version 14.0 (StataCorp, College Station, TX, USA) and AMOS 21.0. *P* values less than 0.05 were considered statistically significant. The use of structural equation modeling (SEM) relies on a number of underlying assumptions. In this section, we describe these assumptions, and the steps we took to ensure that our model adhered to these standard specifications.

First, SEMs assume that all missing data is random rather than systematic. In our model, we used t or χ^2^ tests (all P values are above 0.05) to compare women’s characteristics across those with and without missing data. We found no statistical difference, illustrating that our missing data are random. Second, SEMs assume a sufficiently large sample size. The literature suggests that a research team has 5 to 10 cases per parameter, including both latent and measurable variables [28]. Following this rule, in our analysis, a minimum 255–510 observations would be needed for the 51 parameters that we estimate. We collected 780 sample observations, which is a larger size that the recommended minimum. Third, SEMs assume homogeneity. Since all women in our study identified with the same ethnic groups and spoke the same language, we assume that our sample observations are homogenous (or representative) of women in the target area. Fourth, SEMs depend on correct model specification. In fact, the model we use in this study is similar to those used in other studies, including research conducted in Nepal, Ethiopian and Kenya [27, 29, 30]. Moreover, during the process of model development, we balanced the need for parsimony and model thoroughness. A stepwise process was performed in finding the final model. First, a full path model including all the assumed potential associations of variables and latent constructs were developed (Fig. 2). Next, when the correlation coefficients had *P* values above 0.05 were eliminated, the model was reassessed as well as adjusted. Finally, these steps were repeated until all of the P values were less than 0.05 and the model’s goodness of fit index fulfilled model evaluation standards. Fifth, SEMs assume multivariate normality and linearity. By nature of our research design, some of the variables in our study did not meet the standard assumptions of multivariate normality and linearity. Fortunately, maximum likelihood estimation can be used in SEMs to handle binary response variables, which negates the need to satisfy the assumptions of multivariate normality and linearity [31]. This is indeed what we did in our study to address these two assumptions.

Finally, we were also concerned about content validity, scale score reliability, and construct reliability. Usually, content validity requires the use of recognized experts to evaluate whether test items assess defined content and citations of the literature [32]. The reliability of scaling variables was assessed using Cronbach’s coefficient alpha (α = 0.77), and is described in more detail above. Construct validity was evaluated by the fitting the theoretical model with the study’s data. The index that measured the fit of the model is shown in Fig. 2 and indicates high construct validity.

Multiple indicators were used to evaluate the fit of the model, including χ^2^/degrees of freedom (χ^2^/df), goodness of fit index (GFI), normed fit index (NFI), comparative fit index (CFI), adjusted goodness of fit index (AGFI), root mean square residual (RMR), and root mean square error of approximation (RMSEA) [28]. The χ^2^ test has traditionally been used to evaluate the hypothesis that the relationships suggested in the model provide a plausible explanation of the data. However, the number of parameters and size of the sample can affect the value, thus χ^2^/df can reflect a stabilized fitting effect. Generally, a smaller χ^2^/df value indicates a better fit, values ranging from 2 to 5 are recommended as good fit indices [33]. GFI is an alternative to the χ^2^ test and explains the variance and covariance of the data through the variance and covariance obtained by the model fitting. Generally, GFI values over 0.90 indicate a good fit, but GFI is affected by the number of variables [33]. AGFI is adjusted by the model’s degrees of freedom and number of parameters, and values for the AGFI also range between 0 and 1 and it is generally accepted that values of 0.90 or greater indicate well fitting models [34]. NFI assesses the model by comparing the χ^2^ value of the model to the χ^2^ value of the null model. Values for this statistic range between 0 and 1, with values greater than 0.90 indicating a good fit [28]. CFI is a revised form of the NFI which can provide accurate analysis even when sample size is small. Values for this statistic range between 0.0 and 1.0 with values closer to 1.0 indicating good fit [34, 35].

### Ethical considerations

Prior to the start of this study, ethical approval was obtained from the Research Ethics Committee of Sichuan University, China (registration number K2014022). Since more than half of the participants are illiterate, oral consent with a handprint on the informed consent form was approved by the Research Ethics Committee. In the field, enumerators read the informed consent form including the study’s objectives, procedures, risks, and benefits for each participant before interviews began. All participants gave oral consent with a handprint on the informed consent form in the study.

## Results

Twenty questionnaires that were missing information were deemed unusable for the study and removed, giving us an efficiency rate of 97% and a total study enrollment of 760 women from 68 villages.

### Basic situation of participants

Table 1 shows the characteristics of the participants and their perceived quality of hospital care, related maternal care knowledge, travel time from home to nearest hospital and use of maternal health services. The average age of participants was 28.83 ± 6.41 years, and only 11.71% of the participants had received a junior high or higher education level. Over 75% of the participants were Yi minority, and less than one-third of them had given birth to their first-born. About 90% of the participants were farmers who lived at home and possessed fixed assets (Fixed assets were measured by possession of water supply, television, refrigerator, air conditioner, water heaters, motorcycle, car, computer, and pumping toilet. The index was calculated by constructing a linear index from asset ownership indicators using principal component analysis to derive weights).

**Table 1:** Basic information and use of maternal health services among participants, Sichuan province, western China, 2014

Variables	Mean ± SD or % (No.)
Participant SES	
Age (years)	28.83 ± 6.41
Junior or higher education level,%	11.71 (89)
Yi minority,%	79.73 (606)
First parity,%	24.47 (186)
Farmer or housewife,%	92.50 (703)
Fixed asset,%	89.47 (680)
Perceived quality of hospital care	
Ability of THC^a^, yes%	71.18 (541)
Equipment of THC, good%	44.08 (335)
Techniques of THC, good%	46.45 (353)
Doctors in THC, good%	54.21 (412)
Sanitation of THC, good%	51.45 (391)
Ability of CH ^b^, yes%	93.03 (707)
Equipment of CH, good%	43.03 (327)
Techniques of CH, good%	43.55 (331)
Doctors in CH, good%	42.24 (321)
Sanitation of CH, good%	44.74 (340)
Knowledge score ^b^	4.68 ± 2.32
Travel time from home to the nearest hospital, > 1 h	14.61 (111)
Maternal health service use	
Antenatal care rate,%	68.94 (524)
Hospital delivery rate,%	48.29 (367)
Postpartum visit rate,%	28.42 (216)

*a*, township health center; *b*, county hospital; *c*, knowledge score on maternal care

While 71.2% of participants believed their local township health center had the ability to provide maternal health services, confidence levels in the centers’ equipment, techniques, doctors, and sanitation levels were lower. The proportion of responses indicating township health centers were “good” was around 50% for each of these four aspects. Rankings of equipment, techniques, doctors, and sanitation levels in county hospitals were even lower, with less than 45% of respondents rating these aspects as “good.” However, 93.0% of participants believed that county hospitals had the ability to provide maternal health services, which was substantially higher than township health centers.

The average score for knowledge on maternal care was 4.68 ± 2.32. In terms of travel time to the nearest hospital, 14.6% of participants had a travel time of more than 1 h to the closest care facility. The rate of ANC use was 68.9%, which was the highest reported rate in comparison to the hospital delivery rate of 48.3% and postpartum visit rate of 28.4%.

### Univariate analysis for use of maternal health services

Table 2 shows univariate analysis results for factors related to the use of maternal health services. *P* values lower than 0.05 were considered statistically significant. ANC, hospital delivery, and postpartum care were all more likely to be used by participants with the following traits: younger age, higher education levels, Han ethnicity, first pregnancy, migrant worker, possesses fixed family assets. Participants who believed the township hospital center doctors had good attitudes or that the county hospitals offered good quality of care were more likely to use ANC services. Participants who believed the township hospital centers and county hospitals had good equipment, techniques and doctor attitudes were more likely to have a hospital delivery, and participants who believed that the equipment and techniques of the township health centers were good were more likely to schedule postpartum visits. A higher maternal care knowledge score and shorter time between home and the nearest hospital also increased rates of both ANC use and hospital delivery; however, these variables had no significant correlation with rates of postpartum care. Finally, ANC use was a positive predictor of both hospital delivery and postpartum care.

**Table 2:** Univariate analysis results of use of maternal health services, Sichuan province, western China, 2014

Variables	Antenatal Care (*n* = 760)	Hospital Delivery (*n* = 760)	Postpartum visits (*n* = 760)
	*χ*^2^/*t*	*χ*^2^/*t*	*χ*^2^/*t*
Age (t-test)	6.22**	7.68**	5.62**
More than junior high school education	36.08**	66.13**	48.01**
Han ethnicity	29.44**	115.98**	101.00**
First child	29.44**	74.68**	33.92**
Migrant worker	10.14**	13.79**	4.31*
Fixed asset score more than 0	17.04**	26.18**	14.91**
THC^a^ can provide services: yes	1.92	0.08	1.44
Equipment of THC good	0.10	4.66*	11.08**
Techniques of THC good	0.32	1.52	3.95*
Doctors in THC good	4.81*	0.08	0.00
Sanitation of THC good	3.21	0.49	0.97
CH^b^ can provide services: yes	0.61	3.53	1.65
Equipment of CH good	12.74**	18.19**	1.72
Techniques of CH good	12.98**	19.50**	1.65
Doctors in CH good	9.76**	13.49**	0.20
Sanitation of CH good	12.67**	24.37**	3.38
Knowledge score (t-test)	−7.73**	−7.88**	−8.30**
Travel time to hospital < 1 h	15.15**	10.28**	1.07
Used ANC	–	94.49**	53.48**

*a*, township health center; *b*, county hospital; *P value < 0.05, **P value < 0.01

### SEM multivariate analysis for use of maternal health services

The basic model was created based on theoretical knowledge and the research hypothesis, and was then modified by maintaining the statistically significant terms and removing the non-significant paths. The adjusted model had a better fit than the basic model, with the following indicators: χ^2^/df = 2.06, GFI = 0.96, NFI = 0.96, CFI = 0.98, AGFI = 0.91, RMR = 0.06 and RMSEA = 0.04. Figure 2 shows both the basic and modified models along with the evaluation indicators for each.

**Fig. 3:**
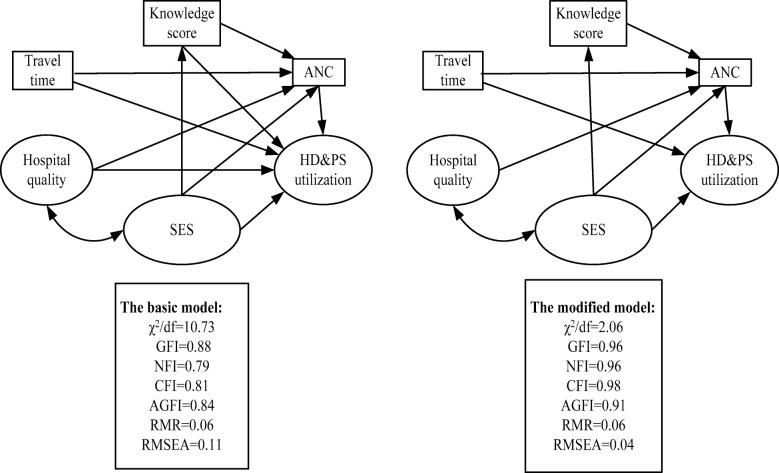
Significant regression path among dependent and independent variables in the structural equation modeling, Sichuan province, western China, 2014. Knowledge score = knowledge score on maternal care. HD&PS utilization = utilization of hospital delivery and postpartum visits. SES = participant socioeconomic status; Hospital Quality = perceived quality of hospital. Travel time = travel time from home to the nearest hospital; THC = township health center. CH = county hospital; e = errors; All the *P* values below the 0.05

According to Fig. 3 and Table 3, SES, perceived quality of hospital care, travel time from home to the nearest hospital, and maternal care knowledge were correlated with the use of ANC services. SES played an essential role in the use of ANC services, with a total effect of − 0.41, direct effect of − 0.36 and an indirect effect through maternal care knowledge score of − 0.05 (calculated by multiplying − 0.43 and 0.12, the correlation coefficients in the path of Fig. 3). Of all the SES indicators, ethnicity was the strongest predictor of ANC use, (*β* = − 0.27; *P* < 0.01 in Table 4). Perceived quality of hospital care was directly associated with ANC with an effect value of 0.10. The perceived quality of township health center equipment, techniques, doctor attitudes, and sanitation contributed the largest to overall perceived quality of care (*β* =0.09; *P* < 0.01 in Table 4). The time from home to nearest hospital was negatively correlated with use of ANC, with an effect of − 0.13. The total effect of pregnancy knowledge score on ANC was 0.12. All data are shown in Tables 3 and 4.

**Table 3:** Direct, indirect and total effects of dominants on use of maternal health services, Sichuan province, western China, 2014

Variables	Total effect		Direct effect		Indirect effect	
	ANC	HD&PS^a^	ANC	HD&PS	ANC	HD&PS
SES^b^	−0.41	−0.81	−0.36	−0.75	−0.05	−0.06
Knowledge^c^	0.12	0.02	0.12	–	–	0.02
Hospital quality^d^	0.10	0.01	0.10	–	–	0.01
Travel time^e^	−0.13	− 0.11	− 0.13	− 0.09	–	− 0.02
ANC	–	0.13	–	0.13	–	–

*a*, use of hospital delivery and postpartum visits; *b*, women’s socioeconomic status

*c*, knowledge score on maternal care; *d*, perceived quality of hospital

*e*, time from home to the nearest hospital with sufficient maternal health services

**Table 4:** Relationship of the measured indicators, latent variables with use of maternal health services, Sichuan province, western China, 2014

Variables	Standardized regression coefficients	
	ANC	HD&PS^a^
SES^b^	0.37	0.66
Age	−0.18 = 0.43 ×-0.43	0.28 = − 0.43 × 0.66
Ethnic	−0.27 = 0.63 ×-0.43	0.42 = 0.63 × 0.66
Education	0.23 = −0.54 ×-0.43	−0.36 = − 0.54 × 0.66
Parity	0.17 = − 0.39 ×-0.43	−0.26 = − 0.39 × 0.66
Occupation	0.09 = 0.22 ×-0.43	0.15 = 0.22 × 0.66
Fixed assets	−0.11 = 0.25 ×-0.43	0.17 = 0.25 × 0.66
Perceived quality of hospital	0.10	–
Ability of THC^c^	0.02 = 0.22 × 0.10	–
Equipment of THC	0.09 = 0.87 × 0.10	–
Technique of THC	0.09 = 0.90 × 0.10	–
Doctor’s attitude of THC	0.09 = 0.91 × 0.10	–
Sanitation of THC	0.09 = 0.91 × 0.10	–
Ability of CH^d^	0.01 = 0.05 × 0.10	–
Equipment of CH	0.02 = 0.21 × 0.10	–
Technique of CH	0.02 = 0.22 × 0.10	–
Doctor’s attitude of CH	0.02 = 0.24 × 0.10	–
Sanitation of CH	0.02 = 0.23 × 0.10	–
Travel time^e^	−0.13	−0.09
Knowledge^f^	0.12	–
ANC	–	0.13

*a*, use of hospital delivery and postpartum service; *b*, women’s social economic status

*c*, township health center; *d*, county hospital; *e*, time from home to the nearest hospital

*f*, knowledge score on maternal care

SES, travel time to the nearest hospital and ANC were directly correlated with hospital delivery and postpartum visit rates, while perceived quality of hospital care and maternal health knowledge had smaller indirect correlations. The total effect of SES was − 0.81, with a direct effect of − 0.75 and an indirect effect (through ANC) of − 0.06 (calculated by multiplying − 0.36 and 0.13, the correlation coefficients in the path of the Fig. 3). Ethnicity was the most powerful predictor among the SES measures of hospital delivery and postpartum visits. The direct effect of travel time to nearest hospital was also significant, with an effect value of − 0.09. ANC was directly associated with hospital delivery and postpartum visit rates with an effect value of 0.13. Perceived quality of hospital care was indirectly associated with hospital delivery and postpartum visit services through ANC use, with an effect value of 0.01 (calculated by multiplying 0.10 and 0.13, the correlation coefficients in the path of the Fig. 3). There was no direct association between maternal care knowledge and hospital delivery and postpartum visit rates, but there was an indirect effect of 0.02 through ANC (calculated by multiplying 0.12 and 0.13, the correlation coefficients in the path of Fig. 3). Figure 3, Table 3, and Table 4 show the details.

In Table 4, each latent variable in the model is broken down into its component parts to examine their relationship with maternal health service use. Among all the measured variables, ethnicity had the strongest correlation with use of ANC, with a correlation coefficient of − 0.27. As for hospital delivery and postpartum visits, educational attainment had the strongest correlation with a correlation coefficient of − 0.36.

## Discussion

Our results show the severity of maternal health care underutilization in rural minority areas of Sichuan province, western China. We found that the rates of ANC services, hospital deliveries, and postpartum visits in our survey areas were 68.9, 48.3, and 28.4%, respectively. These findings are consistent with a 2012 study of minority women in Sichuan province which reported hospital delivery rates below 50% [17]. Other studies of maternal health care in both China and India have shown a large utilization gap between urban and rural regions [36, 37]. However, the rates in our study area were notably lower than rural China region averages obtained by the 5th National Health Services Survey in 2013, which were 97.3, 96.8, and 54.3%, respectively [10]. Low maternal care utilization rates in rural areas indicate a critical public health problem, and identifying the factors related to the use of maternal health services in poor rural areas is essential to solving this problem.

Through our model, we found that all three types of maternal health care were either directly or indirectly impacted by participants’ perception of health care quality, maternal care knowledge, SES, and travel time from home to the nearest hospital. By examining ANC as an independent variable, we found that ANC was positively correlated with both hospital delivery and postpartum care rates. Frequent ANC visits have also been identified as a positive predictor of hospital delivery in rural areas of other developing countries, which may be because positive health interactions with doctors make women more inclined to continue using maternal health services [29, 38]. Despite this relationship, ANC has rarely been studied as an intermediate variable in maternal health care. Previous studies have predominantly focused on factors that independently affect the three types of maternal health service use [38–40]. Thus ignoring ANC as an intermediate variable affecting hospital delivery and postpartum visit rates. Our research can begin to fill this gap by revealing how measures focused on increasing the rates of ANC use can in turn increase rates of hospital delivery and postpartum care. Since ANC use rates are highly variable and influenced by multiple factors [41, 42], studying ANC is particularly useful in illuminating possibilities for improving maternal health care use rates. For instance, maternal care knowledge and perceived quality of health care were not directly correlated with the use of hospital delivery and postpartum visits; however, by using SEM we found that these variables indirectly affect postpartum visit rates through their impact on ANC rates.

Our findings indicate that perceived quality of health care is an important factor contributing to whether women use maternal health care services. This is consistent with the studies in primary health care research and skilled delivery service research, which have shown that understanding a population’s perceptions of quality of medical care is critical to developing measures that will increase the use and accessibility of primary health care or maternity services [30, 43]. Among the aspects of care that we asked participants to evaluate, perceptions of the township health center contributed most to the perception of hospital quality of care. Thus efforts to increase maternal health care use rates should focus on improving public perception of township health services. This could include outreach and educational measures as well as measures to improve doctor practice and equipment at township health centers.

Low pregnancy knowledge was also related to lower use rates of maternal health care [44]. The use of maternal health services was increased with an increase in health knowledge level. This indicates that education about maternal health care has the potential to increase use rates. Our study also revealed that pregnancy knowledge was lower among participants who had inferior SES, indicating that effective maternal health education programs should be designed to target women of this demographic.

The results of this study also indicated that travel time from home to the nearest hospital is an important factor for all three types of maternal health services, which was the same barrier in other rural areas of developing counties [22, 45]. The tough and mountainous environment of the rural areas in our study as well as lack of transportation options remain a barrier for women seeking maternal health services. Geographic accessibility should thus be a focus in efforts to increase maternal health services use in poor, rural minority regions of western China. Such efforts could include expanding transportation options, increasing the number of care centers, or implementing mobile clinics.

Geographic accessibility also may differ for a single participant depending on the circumstance. As noted above, our variable of travel time does not account for availability of transportation, and it is worth noting that transportation options may be different for women considering ANC, hospital deliveries, or postpartum care. For instance, public transportation or carpools are much easier to schedule in advance for ANC or postpartum care than for hospital deliveries. For the many women in our survey who travel to the hospital by foot, transportation is also more difficult for hospital deliveries or postpartum visits, since women who are about to give birth or who need to carry an infant will be less physically able to travel long distances by foot. Thus, comprehensive solutions to underutilization of maternal health care also need to consider transportation concerns that may be rather unique to rural women seeking maternal health care. This might include solutions such as expanded public transportation or public transportation with flexible times, mobile midwives equipped with their own transportation tools, or town-based accommodation prior to birth.

### Limitations

Since we conducted the survey in the areas with the township health centers to provide maternal care services, the areas without the capability of providing maternal care services had not been explored. The attitude about use of maternal health services from surrounding population that may be an encouraging environment factor was not included in our survey. Despite the limitations, the study has shed the light on the effect of ANC for promoting the whole utilization of maternal care services among the rural Yi minority areas.

### Conclusions

Improving ANC behavior should be a priority of maternal health care reforms in poor, rural Yi minority regions in western China, since ANC is an intermediate variable that can in turn affect other types of maternal care. Maternal health care use rates can be increased through pregnancy education programs targeted toward women with lower pregnancy knowledge, and through enhancing the services provided by township health centers. However, given the long travel times and few transportation tools available to the women in our study, increasing ANC rates and pregnancy knowledge are likely necessary but insufficient methods to increase maternal health care usage overall. Lack of physical accessibility to medical services is also a large barrier to maternal health care access and improving accessibility should be a focus of reforms.

## Additional file

Additional file 1: Questionnaire for the maternal health services survey. The questionnaire is divided into four parts: (1) The basic information of women; (2) Perceived quality of health care and travel time to the nearest hospital; (3) Women’s knowledge on maternal care; (4) Utilization of maternal health services. (DOCX 19 kb)

## Abbreviations

ANC: Antenatal care
CH: County hospital
HD&PS utilization: Hospital delivery and postpartum visits
SEM: Structural equation modeling
SES: Social economic status
THC: Township health center
