# Supplementary material for: Use of maternal health services among women in the ethnic rural areas of western China
Source: BMC Health Serv Res. 2019 Mar 19;19:179. doi: 10.1186/s12913-019-3996-2 (PMC6425603; doi:10.1186/s12913-019-3996-2)
Supplement: Supplementary file 1 — Questionnaire for the maternal health services survey. The questionnaire is divided into four parts: (1) The basic information of women; (2) Perceived quality of health care and travel time to the nearest hospital; (3) Women’s knowledge on maternal care; (4) Utilization of maternal health services. (DOCX 19 kb) [file 12913_2019_3996_MOESM1_ESM.docx]

**Additional File 1: Questionnaire for the Maternal Health Services Survey**

CODE：□□□□□□□□

**Part 1 Basic information**

1. What’s your age (calculated by the Chinese lunar calendar)? _____
2. What’s your ethnicity? _____

A=Han B=Yi C=Hui D=Tibetan E=Naxi

F=Mongolian G=others, please explain _____

1. What is your highest level of education? _____

A= illiterate B= primary school C= junior/middle school

D=high school (academic or vocational) E= university or above

1. What’s your occupation? _____

A=farming B=employee C=self-employed business

D=working in government departments E=others, please explain _____

1. What’ your child’s age? _____months _____days
2. What’s your child’s gender? _____

A=male B= female

1. What’s the parity of this child? _____(number)
2. The family fixed asset:
3. Does your family have the running water? _____ A=yes B=no
4. Does your family have a television? _____ A=yes B=no
5. Does your family have a refrigerator?_____ A=yes B=no
6. Does your family have an air conditioner? _____ A=yes B=no
7. Does your family have a water heater? _____ A=yes B=no
8. Does your family have a motorcycle or electric bike?_____ A=yes B=no
9. Does your family have a car or truck?_____ A=yes B=no
10. Does your family have a computer? _____ A=yes B=no

**Part 2** **Perceived quality of health care and travel time to the nearest hospital**

1. Do you think the county hospital can provide maternal health services? _____

A= yes B=no

1. What do you think of the equipment of the county hospital? _____

A=good B=average C=bad

1. What do you think of the techniques of the county hospital? _____

A=good B=average C=bad

1. What do you think of the doctor’ attitude of the county hospital? _____

A=good B=average C=bad

1. What do you think of the sanitation of the county hospital? _____

A=good B=average C=bad

1. Do you think the township health center can provide maternal health services? _____

A= yes B=no

1. What do you think of the equipment of the township health center? _____

A=good B=average C=bad

1. What do you think of the technique of the township health center? _____

A=good B=average C=bad

1. What do you think of the doctor’ attitude of the township health center? _____

A=good B=average C=bad

1. What do you think of the sanitation of the township health center? _____

A=good B=average C=bad

1. What’s your travel time to the nearest hospital capable of providing maternal health services? _____ minutes

**Part 3** **Knowledge on maternal care**

1. Do you think that pregnant women who have physical contact with cats and dogs would be in the risk of miscarriage? _____

A=yes B=no

1. Do you think that pregnant women need to go to the hospital in time when they have vaginal bleeding? _____

A=yes B=no

1. Do you think that pregnant women need to go to the hospital in time when they have a bellyache? _____

A=yes B=no

1. Do you think that pregnant women need to go to the hospital in time when they have lower limb edema? _____

A=yes B=no

1. Do you think that pregnant women need to go to the hospital in time when they have irregular fetal movement? _____

A=yes B=no

1. Do you think that antenatal care is necessary for pregnant women? _____

A=yes B=no

1. What’s the recommended number of antenatal care for pregnant women? _____

A=one time B=twice C=three times D four times E five times and more

1. Do you think the postpartum visit is necessary for women and their children? _____

A=yes B=no

**Part 4** **Utilization of maternal health services**

1. For this child, have you ever used the antenatal care service? _____

A=yes B=no

1. For this child, where was she/ he born? _____

A=home B=hospital C=others, please explain_____

1. For this child, have you ever used the postpartum visits service? _____

A=yes B=no
